# Ten-year experience with ophthalmic artery chemosurgery: Ocular and recurrence-free survival

**DOI:** 10.1371/journal.pone.0197081

**Published:** 2018-05-23

**Authors:** Jasmine H. Francis, Ariana M. Levin, Emily C. Zabor, Y. Pierre Gobin, David H. Abramson

**Affiliations:** 1 Memorial Sloan-Kettering Cancer Center, New York, New York, United States of America; 2 Weill Cornell Medical Center, New York, New York, United States of America; Massachusetts Eye & Ear Infirmary, Harvard Medical School, UNITED STATES

## Abstract

**Purpose:**

To report associations between disease- and treatment-related variables and rates of recurrence-free survival and ocular survival in eyes treated with ophthalmic artery chemosurgery (OAC) for retinoblastoma.

**Design:**

Pre-post study.

**Subjects:**

All eyes treated with OAC for retinoblastoma at Memorial Sloan Kettering Cancer Center between May 2006 and February 2017.

**Methods:**

This retrospective review included 452 retinoblastoma eyes treated with OAC. The Kaplan-Meier method was used to estimate recurrence-free survival (RFS), progression-free survival (PFS) and ocular survival (OcS), and Cox regression was used to estimate hazard ratios. Eyes treated in the pre-intravitreous chemotherapy era were analyzed separately from eyes treated in the intravitreal era.

**Main outcome measures:**

Recurrence-free survival, ocular survival, associations with risk of recurrence

**Results:**

Disease and treatment characteristics were recorded over a median 23.6 month follow-up. One-year OcS, PFS and RFS were 96% (95% CI 93–99%), 88% (95% CI 88–94%) and 74% (95% CI 67–81%) in the pre-intravitreal era and 96% (95% CI 94–99%), 93% (95% CI 89–96%) and 78% (95% CI 72–83%) in the intravitreal era, respectively. Presence of vitreous seeds was associated with increased risk of recurrence in the pre-intravitreal era but not in the intravitreal era. Longer time interval between OAC sessions was associated with increased risk of recurrence and majority OAC access via the ophthalmic artery was associated with decreased risk of recurrence in both eras.

**Conclusions:**

Approximately a quarter of eyes initially treated with ophthalmic artery chemosurgery develop recurrent disease, with the majority of recurrences within the first year following completion of OAC. Despite this, these eyes have a very good chance of salvage. In eyes with vitreous seeds at presentation, intravitreal injections are useful in minimizing future vitreous recurrence. Eyes that receive the majority of drug infusions via non-ophthalmic artery routes or greater interval between OAC are more likely to recur and might warrant closer monitoring.

## Introduction

Although retinoblastoma is the pediatric solid cancer with the highest survival rate in developed countries (>99% in some series), this was historically accomplished by removing one or both eyes. [1] For most of the 20^th^ century, the only way to salvage eyes with advanced intraocular disease was external beam irradiation. [2] Radiation did salvage 25% of such eyes and often with useful vision, but at least a quarter required additional, subsequent focal treatments. [3] It is impossible to know from the published literature if this was because of persistent disease or local recurrences, because such information was not tabulated. In some cases, additional treatment was for “new” tumors, which were not thought to be recurrences.

In the 1990s, radiation was abandoned in favor of multiagent systemic chemotherapy. Overall, only 36.7% were salvaged with intravenous chemotherapy. [4] Approximately 29% of Group D eyes were primarily enucleated. [5] Ninety-nine percent of eyes treated with systemic chemotherapy required additional laser, cryotherapy, brachytherapy or external beam irradiation (and even enucleation), but it is impossible to know how many of these were “routine” additional treatments and how many were treated because of local recurrences. In fact, there is no existing definition of “recurrence” in the retinoblastoma literature.

The introduction of intra-arterial chemotherapy (ophthalmic artery chemosurgery, OAC) in 2006 allowed the majority of advanced eyes to be salvaged. [6] Within five years of its introduction, many centers worldwide were using it as first-line treatment for advanced eyes. [7] With time and experience, a majority of eyes treated with OAC were salvaged without compromising patient survival or vision. [8] Some blind eyes even regained visual potential after OAC. [9]

The purpose of this study is to define and characterize local ocular recurrences after OAC in retinoblastoma eyes. Specifically, this study aims to report the rate of ocular recurrence requiring additional treatments, the timing and nature of recurrences, whether the route of administration (internal or external carotid artery) influenced success, the impact of intravitreous chemotherapy on ocular salvage, and the impact of delays in therapy on success, in addition to reporting on patient and ocular survival in the largest series of OAC patients worldwide (with the longest follow-up) from Memorial Sloan Kettering Cancer Center (MSKCC)

## Methods

This is a retrospective, single institution review of all retinoblastoma eyes treated with ophthalmic artery chemosurgery at MSKCC between May 2006 and February 2017. The MSKCC institutional review board approved this study and waived the requirement for an informed consent. This study was conducted in accordance with the Declaration of Helsinki.

### Eyes and treatments

Eyes were included if they were treated for retinoblastoma at MSKCC with OAC and completed OAC treatment on or before November 2016 (allowing for 3-month follow-up after treatment completion through February 2017). Exclusion criteria were loss to follow-up within 3 months of treatment end or enucleation within 3 months of the last OAC session for reasons other than progression of disease (e.g. painful blind eye without progression of disease). Four hundred and ninety-nine eyes fit the inclusion criteria. Forty-seven eyes were excluded due to inadequate follow-up or because they had not yet completed treatment at the study end date. Eyes that initiated OAC treatment but subsequently underwent enucleation due to disease progression (with no treatment-free period) were categorized as treatment failure.

Eyes were divided into two eras. Eyes that ended the OAC treatment course in the pre-intravitreous chemotherapy era (through February 2013) were analyzed separately from eyes that ended treatment in the intravitreal era (after February 2013).

Eyes were classified according to Reese-Ellsworth (RE) and the Children’s Oncology Group version of the International Classification of Retinoblastoma (ICRB). Patients were defined to have germline mutations if they had positive family history, bilateral disease, or mutation confirmed by genetic testing. Eyes were defined as non-naive if they underwent treatment (EBR, systemic chemotherapy, intra-arterial chemotherapy) at an outside hospital. Three additional eyes were categorized as non-naive, because they underwent prior treatment courses at MSKCC that ended more than 3 months before the OAC treatment course. Bridge chemotherapy was defined as chemotherapy at MSKCC, typically single agent carboplatin, given to an infant intended to have OAC but too small or young at first visit. “International bridge” was defined as chemotherapy given to an infant abroad who was intended to start treatment at MSKCC, but was delayed due to international travel (e.g. obtaining a visa) and thus underwent systemic chemotherapy during the delay. OAC method was previously described. [10]

Twenty-three of 166 eyes (14%) received bridge therapy in Era 1 (median 3 infusions) with 18 eyes receiving carboplatin (single agent), 4 receiving vincristine and carboplatin (VC), and 1 receiving vincristine, etoposide and carboplatin (VEC). Twenty-one of 270 eyes (7.8%) received bridge therapy in Era 2 (median 2 infusions) with 18 eyes receiving carboplatin, and 1 each receiving VC, CE, or VEC. Chemotherapy agents were determined by the medical oncologist based on initial response to carboplatin, extent of disease, or other individual factors.

### Outcome data

**Fig 1:**
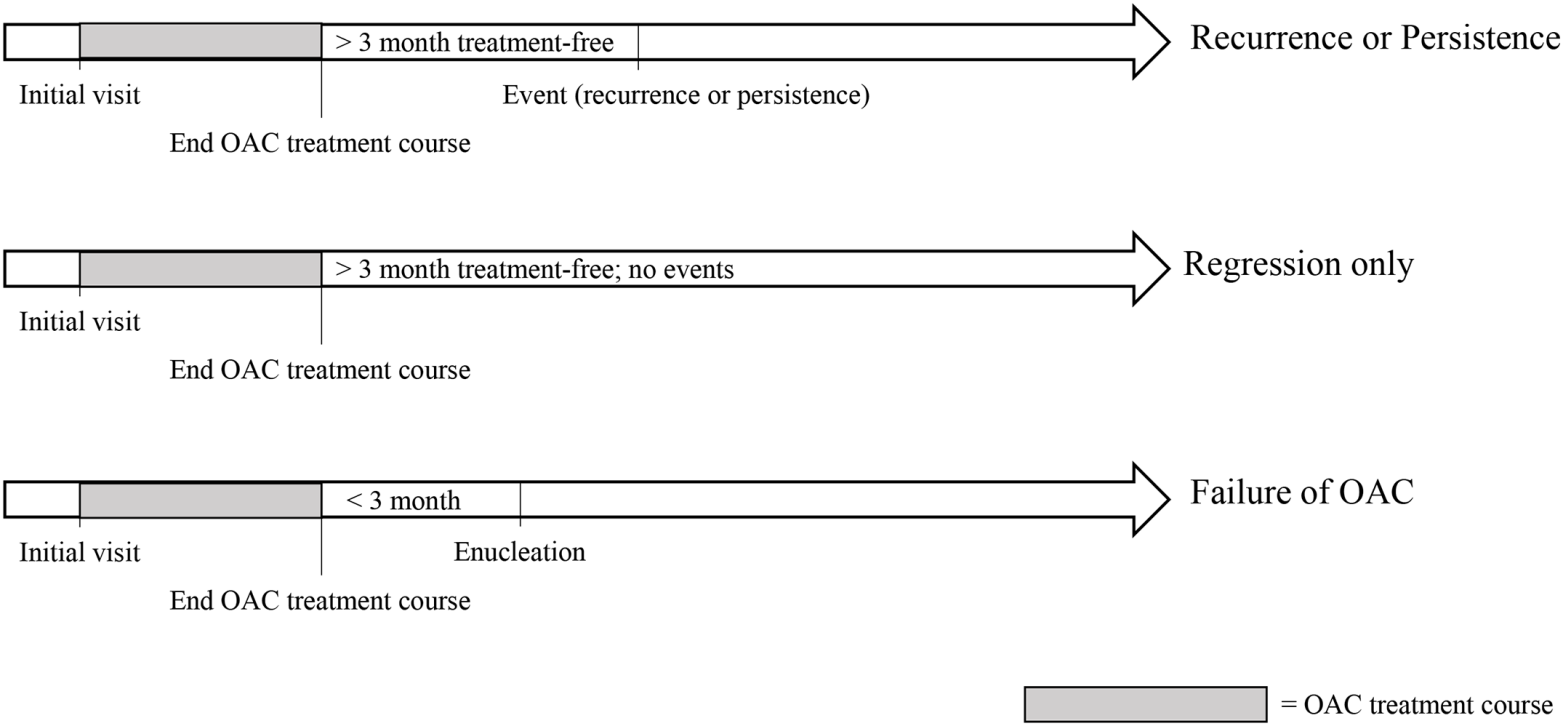
Time courses for event classification.

The OAC treatment course was defined as a period that included OAC and adjunctive treatments without a treatment-free period lasting 3 months or more. By definition, the OAC treatment course was bookended by treatment-free periods lasting at least 3 months. For each case that underwent treatment more than 3 months after completing the OAC treatment course, the reason for treatment was categorized as one of two mutually exclusive events: recurrent disease or persistent disease. (Fig 1) Recurrence was defined as new tumor activity visible on ophthalmoscopic exam, including regrowth from regressed tumor and/or new subretinal or vitreous seeds. If ophthalmoscopic exam was obscured, recurrence included new activity detected on ultrasound or MRI. Regrowth detected on optical coherence tomography (OCT), but not ophthalmoscopic exam, was not categorized as recurrence, because lack of OCT data for early patients would introduce time bias. Persistence was defined as stable, non-calcified tumor that demonstrated no new growth but was treated at the clinician’s discretion. Primary outcomes were recurrence, persistence, and enucleation. Secondary outcomes included metastasis, secondary cancers, and deaths.

### Statistics

Categorical variables were summarized using frequency and percent; continuous variables were summarized using median and range. Between-group comparisons were made using generalized estimating equations models with sandwich variance estimates and exchangeable correlation structure, to account for the correlation between multiple observations (i.e. left eye and right eye) from a single patient.

Statistical analyses were conducted using R software version 3.2.5 (R Core Development Team. Vienna, Austria) including the ‘survival’ package. The Kaplan-Meier method estimated recurrence-free survival (RFS), treatment-free survival (TFS), and ocular survival (OcS). For RFS analysis, event was defined as recurrence. RFS time was calculated from the end date of the OAC treatment period to date of diagnosis of recurrent disease. Patients with persistent disease were excluded from analysis of RFS. For TFS analysis, event was defined as treatment for recurrent or persistent disease. TFS time was calculated from the end date of the OAC treatment period to the start date of treatment for recurrent or persistent disease. Patients with less than 3 months of follow-up time were excluded from analysis of RFS and TFS. PFS was defined as time to enucleation, external beam radiation, or OAC. Follow-up time for PFS started at the date of first visit and continued to the date of the first specified event (and therefore included patients who failed initial treatment). For ocular survival (OcS) analysis, event was defined as enucleation. Patients were censored at loss to follow-up or study end (February 28, 2017).

Cox regression with robust variance estimates was used to obtain p-values for between group comparisons that account for the correlation between multiple observations from a single patient. A p-value < 0.05 was considered statistically significant.

## Results

### Patient, treatment, and eye characteristics

**Fig 2:**
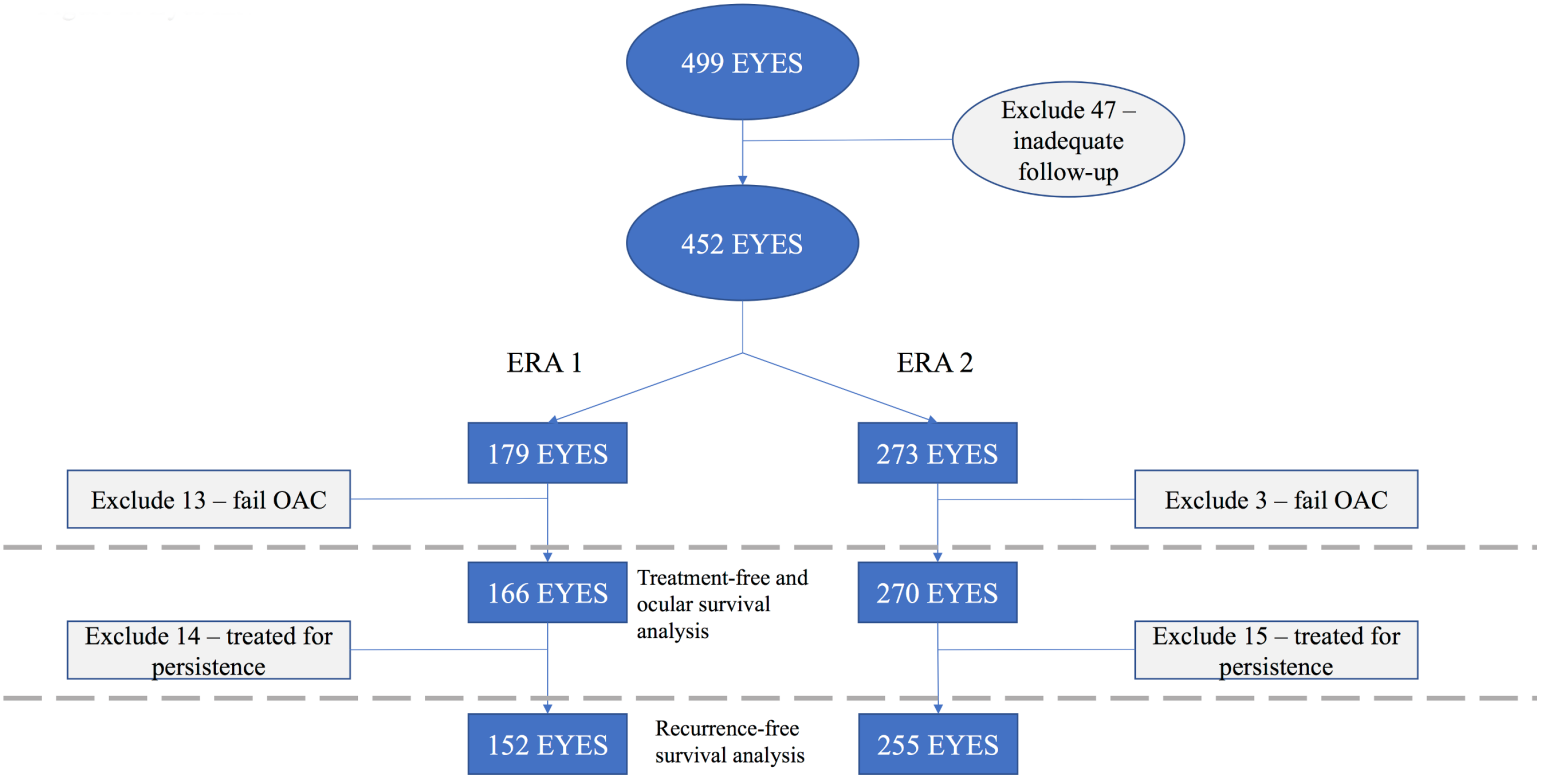
Eyes included in analyses.

A total of 452 eyes were included in this study. (Fig 2) Of these, 16 eyes (3.5%) failed treatment in the OAC treatment course and were excluded from analyses. Thirteen eyes failed in Era 1; three eyes failed in Era 2 (7.3% versus 1.1%, p-value 0.001). Thus, analyses included 436 eyes: 166 (38.1%) from Era 1 and 270 (61.9%) from Era 2.

Compared with Era 1, a larger percentage of Era 2 eyes were treatment-naive (34.9% versus 63%, p-value <0.001), and a larger percentage were hypertensive on first visit (7.8% versus 35.6%, p-value 0.002). (Table 1)

**Table 1:** Eye and disease characteristics. Numbers shown are median (min, max) for continuous variables and N (%) for categorical variables.

	Overall (N = 436)	Era 1 (N = 166)	Era 2 (N = 270)	p-value
Age at first visit (months)	13.4 (0.1, 195.1)	11.1 (0.3, 195.1)	14.8 (0.1, 122)	0.92
Age at last treatment (months)	16.8 (3.8, 196.9)	15.9 (3.8, 196.9)	17.8 (5.3, 124.5)	0.462
Weight (kg) at first OAC^a^	10 (4.5, 71)	10 (4.5, 71)	10.5 (5.4, 35.9)	0.112
Gender				0.123
Female	219 (50.2)	92 (55.4)	127 (47)	
Male	217 (49.8)	74 (44.6)	143 (53)	
Family history				0.256
No	387 (88.8)	143 (86.1)	244 (90.4)	
Yes	49 (11.2)	23 (13.9)	26 (9.6)	
Laterality				< .001
Bilateral	271 (62.2)	114 (68.7)	157 (58.1)	
Unilateral	165 (37.8)	52 (31.3)	113 (41.9)	
Side				0.781
Left	215 (49.3)	83 (50)	132 (48.9)	
Right	221 (50.7)	83 (50)	138 (51.1)	
RE^b^ class				0.507
1	11 (2.5)	6 (3.6)	5 (1.9)	
2	24 (5.5)	13 (7.8)	11 (4.1)	
3	44 (10.1)	14 (8.4)	30 (11.1)	
4	27 (6.2)	12 (7.2)	15 (5.6)	
5	279 (64)	104 (62.7)	175 (64.8)	
NA	51 (11.7)	17 (10.2)	34 (12.6)	
ICRB^c^ class				< .001
A	3 (0.7)	0 (0)	3 (1.1)	
B	42 (9.6)	20 (12)	22 (8.1)	
C	45 (10.3)	18 (10.8)	27 (10)	
D	208 (47.7)	85 (51.2)	123 (45.6)	
E	85 (19.5)	28 (16.9)	57 (21.1)	
NA	53 (12.2)	15 (9)	38 (14.1)	
Vitreous seeds at first visit				0.7
No	169 (38.8)	57 (34.3)	112 (41.5)	
Yes	211 (48.4)	71 (42.8)	140 (51.9)	
NA	56 (12.8)	38 (22.9)	18 (6.7)	
Subretinal seeds at first visit				0.265
No	67 (15.4)	28 (16.9)	39 (14.4)	
Yes	233 (53.4)	72 (43.4)	161 (59.6)	
NA	136 (31.2)	66 (39.8)	70 (25.9)	
Germline mutation				0.881
Negative	94 (21.6)	31 (18.7)	63 (23.3)	
Positive	293 (67.2)	119 (71.7)	174 (64.4)	
NA	49 (11.2)	16 (9.6)	33 (12.2)	
Treatment naive				< .001
No	208 (47.7)	108 (65.1)	100 (37)	
Yes	228 (52.3)	58 (34.9)	170 (63)	
Intraoperative pressure hypertensive				0.002
No	241 (55.3)	90 (54.2)	151 (55.9)	
Yes	109 (25)	13 (7.8)	96 (35.6)	
NA	86 (19.7)	63 (38)	23 (8.5)	

^a^OAC = ophthalmic artery chemosurgery

^b^RE = Reese-Ellsworth

^c^ICRB = International Classification of Retinoblastoma

Mean cumulative and maximum carboplatin doses, as well as cumulative topotecan doses, were increased in Era 2. Era 1 eyes had a higher frequency of adjunctive cryotherapy and plaque. By definition, only Era 2 eyes received intravitreal injections. (Table 2)

**Table 2:** Treatment characteristics. Numbers shown are median (min, max) for continuous variables and N (%) for categorical variables.

	Overall (N = 436)	Era 1 (N = 166)	Era 2 (N = 270)	p-value
Number of primary OAC^a^ infusions	3 (1, 9)	3 (1, 9)	3 (1, 7)	0.081
Maximum melphalan dose	4 (0, 42)	4 (0, 7.5)	4 (0, 42)	0.4
Maximum topotecan dose	0.5 (0, 40)	0.3 (0, 2)	1 (0, 40)	NA
Maximum carboplatin dose	40 (0, 100)	0 (0, 75)	50 (0, 100)	0.001
Cumulative melphalan dose	9 (0, 54)	9 (0, 37.5)	9 (0, 54)	0.09
Cumulative topotecan dose	1.5 (0, 46)	0.6 (0, 8)	2 (0, 46)	0.003
Cumulative carboplatin dose	80 (0, 450)	0 (0, 285)	100 (0, 450)	0.001
OAC^a^ interval (weeks)	28.5 (13.5, 269)	28 (16, 105)	29 (13.5, 269)	0.529
Greater than 3 infusions				0.878
No	101 (23.2)	40 (24.1)	61 (22.6)	
Yes	333 (76.4)	125 (75.3)	208 (77)	
NA	2 (0.5)	1 (0.6)	1 (0.4)	
Greater 50% OAC				0.264
No	103 (23.6)	29 (17.5)	74 (27.4)	
Yes	333 (76.4)	137 (82.5)	196 (72.6)	
Primary periocular				0.299
No	416 (95.4)	164 (98.8)	252 (93.3)	
Yes	20 (4.6)	2 (1.2)	18 (6.7)	
Primary plaque				0.041
No	423 (97)	158 (95.2)	265 (98.1)	
Yes	12 (2.8)	8 (4.8)	4 (1.5)	
NA	1 (0.2)	0 (0)	1 (0.4)	
Primary EBR^b^				1
No	435 (99.8)	165 (99.4)	270 (100)	
Yes	1 (0.2)	1 (0.6)	0 (0)	
Primary/concurrent laser treatment				0.426
No	151 (34.6)	50 (30.1)	101 (37.4)	
Yes	285 (65.4)	116 (69.9)	169 (62.6)	
Primary/concurrent cryotherapy				0.02
No	313 (71.8)	96 (57.8)	217 (80.4)	
Yes	123 (28.2)	70 (42.2)	53 (19.6)	
Bridge				0.383
No	310 (71.1)	143 (86.1)	167 (61.9)	
Yes	44 (10.1)	23 (13.9)	21 (7.8)	
NA	82 (18.8)	0 (0)	82 (30.4)	

^a^OAC = ophthalmic artery chemosurgery

^b^EBR = external beam radiation

### Ocular survival

Median follow-up time among those who did not have enucleation was 26.5 months (minimum (min) = 0, maximum (max) = 119.7). During that time, 33 eyes had an enucleation. Estimates of 1-year ocular survival (OcS) were 96% (95% confidence interval (CI) 93–99%) in Era 1 and 96% (95% CI 94–99%) in Era 2 (p-value 0.629). Variables associated with significantly **increased** risk of enucleation in Era 1 included older age at first visit, older age at last treatment, higher maximum melphalan dose, and higher cumulative melphalan dose. (Table 3) Confirmed germline mutation was the only variable associated with significantly **decreased** risk of enucleation. Confirmed germline mutation was associated with younger age at first visit, lower RE classification, and lower ICRB classification (all p-values < 0.001). In Era 2, treatment-naivety was the only variable associated with significantly **increased** risk of enucleation. No variables were associated with significantly **decreased** risk of enucleation in Era 2.

**Table 3:** Univariable Cox regression analysis for ocular survival.

	Era 1		Era 2	
	HR^a^ (95% CI^b^)	p-value	HR^a^ (95% CI^b^)	p-value
Age at first visit (months)	1.02 (1.00–1.03)	0.005	1.01 (0.99–1.03)	0.224
Age at last treatment (months)	1.02 (1.01–1.03)	0.004	1.01 (0.99–1.03)	0.427
Weight (kg) at first OAC	1.01 (0.98–1.04)	0.514	1.05 (0.97–1.14)	0.219
Gender		0.877		0.979
Female	1		1	
Male	0.93 (0.37–2.35)		1.01 (0.35–2.91)	
Laterality		0.064		0.461
Bilateral	1		1	
Unilateral	2.38 (0.95–5.96)		1.47 (0.53–4.10)	
Side		0.295		0.949
Left	1		1	
Right	0.61 (0.24–1.54)		0.97 (0.35–2.67)	
RE^c^ class		0.063		0.274
1 to 4	1		1	
5	6.9 (0.90–52.64)		3.16 (0.40–24.80)	
Vitreous seeds at first visit		NA		0.059
No			1	
Yes	NA		4.25 (0.95–19.07)	
Germline mutation		0.01		0.956
Negative	1		1	
Positive	0.29 (0.11–0.74)		0.96 (0.27–3.44)	
Treatment naive		0.443		0.012
No	1		1	
Yes	1.44 (0.56–3.69)		6.04 (1.48–24.66)	
Intraoperative pressure hypertensive		0.762		0.171
No	1		1	
Yes	0.73 (0.10–5.43)		2.09 (0.73–6.02)	
Maximum melphalan dose	1.41 (1.09–1.84)	0.009	0.98 (0.85–1.13)	0.768
Maximum topotecan dose	1.24 (0.35–4.37)	0.743	0.99 (0.92–1.07)	0.86
Maximum carboplatin dose	1.01 (0.98–1.03)	0.6	1.02 (0.98–1.06)	0.282
Cumulative melphalan dose	1.07 (1.02–1.11)	0.002	1.00 (0.93–1.07)	0.953
Cumulative topotecan dose	1.16 (0.88–1.52)	0.29	1.02 (0.97–1.08)	0.376
Cumulative carboplatin dose	1.00 (0.99–1.01)	0.943	1.00 (1–1.01)	0.672
OAC^d^ interval (weeks)	1.00 (0.98–1.03)	0.76	0.97 (0.90–1.06)	0.523
Greater than 3 infusions		0.179		0.914
No	1		1	
Yes	2.67 (0.64–11.22)		0.93 (0.26–3.30)	
Greater 50% OA^d^		0.507		0.277
No	1		1	
Yes	1.65 (0.38–7.21)		0.56 (0.20–1.59)	
First treatment OA^d^		0.548		0.071
No	1		1	
Yes	1.82 (0.26–12.81)		0.38 (0.13–1.08)	
Bridge		0.79		
No	1			
Yes	1.19 (0.34–4.15)			
International Bridge		NA		0.135
No			1	
Yes	NA		2.26 (0.78–6.60)	

^a^HR = hazard ratio

^b^CI = confidence interval

^c^RE = Reese-Ellsworth

^d^OAC = ophthalmic artery chemosurgery/ OA = ophthalmic artery

Including treatment failures (i.e. failed initial OAC) as an enucleation event, estimates of 1-year ocular survival (OcS) were 94% (95% CI: 90–98%) in Era 1 and 92% (95% CI: 88–97%) in Era 2 (p-value 0.538).

### Event-free survival (EBR or enucleation)

Event-free survival, defined as time to external-beam radiation (EBR) or enucleation, was calculated for Era 1 only; no patients underwent EBR in Era 2. Median follow-up time was 59.1 months (min = 0, max = 119.7). During this time, 19 eyes had EBR or enucleation. The estimate of 1-year event-free survival was 95.7% (95% CI 92.6–98.9%).

### Progression-free survival (EBR, enucleation or OAC)

Median follow-up time was 25.8 months (min = 3.2, max = 119.7). During this time, 72 patients had a PFS event. Progression-free survival (event defined as EBR, enucleation, or OAC after initial treatment; time starts at initial visit) at one year was 88% (95% CI 83–93%) in Era 1 and 93% (95% CI 89–96%) in Era 2.

### Recurrence-free survival

There were 407 eyes eligible for analyses of recurrence-free survival (RFS). Median follow-up time among those who did not experience a recurrence was 23.6 months (min = 3.2, max = 119.7). During this time, 111 eyes experienced a recurrence. Overall estimates of RFS were 76.3% (95% CI 72.1–80.7%) at 1 year and 70% (95% CI 65.3–75.1%) at 2 years.

**Fig 3:**
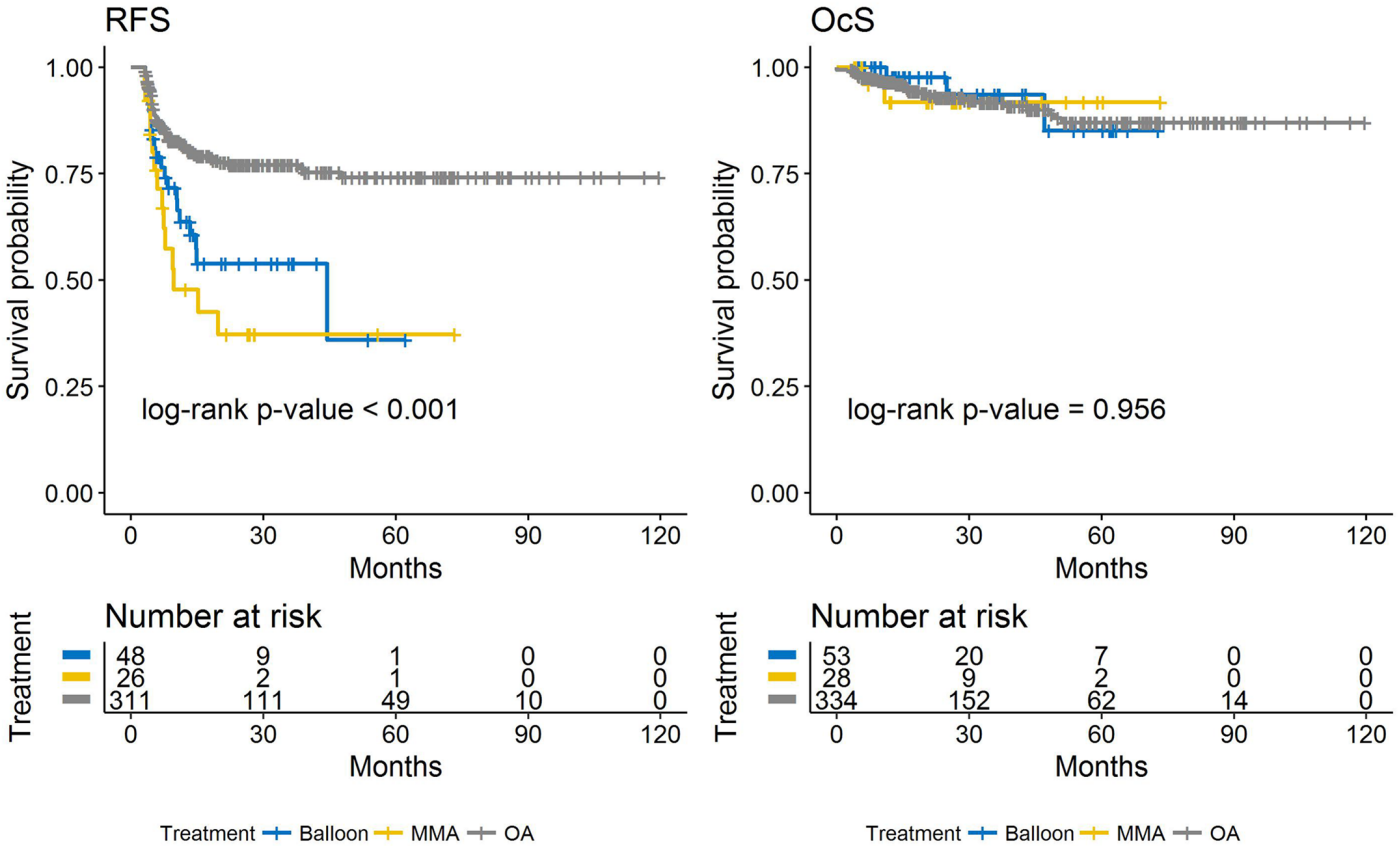
Kaplan-Meier estimates of recurrence-free survival (left) and ocular survival (right) by route of OAC sessions.

Stratified by era, estimates of 1-year RFS were 74% (95% CI 67–81%) in Era 1 and 78% (95% CI 72–83%) in Era 2 (p-value 0.495). Variables associated with significantly **increased** risk of recurrence in Era 1 include older age at first visit, older age at last treatment, higher cumulative melphalan dose, longer time interval between OAC sessions, presence of vitreous seeds at first visit, increased number of drugs used in combination, and bridge chemotherapy. (Table 4) Route of OAC sessions (>50% via ophthalmic artery) was the only variable associated with significantly **decreased** risk of recurrence in Era 1. In Era 2, longer time interval between OAC sessions is the only variable associated with significantly **increased** risk of recurrence. Variables associated with significantly **decreased** risk of recurrence in Era 2 include route of OAC sessions (>50% via ophthalmic artery) and route of first OAC session via the ophthalmic artery. (Fig 3) There was an association between route of OAC sessions and route of first OAC session (p-value <0.001). There was no difference in RFS between patients who underwent the majority of OAC sessions via the middle meningeal artery versus with a balloon.

**Table 4:** Univariable Cox regression analysis for recurrence-free survival.

	Era 1		Era 2	
	HR^a^ (95% CI^b^)	p-value	HR^a^ (95% CI^b^)	p-value
Age at first visit (months)	1.01 (1.00–1.01)	0.01	0.99 (0.98–1.01)	0.489
Age at last treatment (months)	1.01 (1.00–1.01)	0.001	1.00 (0.98–1.01)	0.522
Weight (kg) at first OAC	1.00 (0.98–1.03)	0.678	0.99 (0.94–1.05)	0.802
Gender		0.345		0.872
Female	1		1	
Male	1.30 (0.75–2.26)		0.96 (0.58–1.58)	
Family history		0.083		0.186
No	1		1	
Yes	1.87 (0.92–3.79)		0.54 (0.22–1.34)	
Laterality		0.164		0.647
Bilateral	1		1	
Unilateral	0.63 (0.33–1.21)		1.12 (0.68–1.86)	
Side		0.955		0.835
Left	1		1	
Right	0.98 (0.57–1.71)		1.05 (0.64–1.74)	
RE^c^ class		0.869		0.758
1 to 4	1		1	
5	1.06 (0.53–2.14)		1.11 (0.58–2.10)	
ICRB^d^ class		0.96		0.399
A/B/C	1		1	
D	0.91 (0.45–1.85)		1.64 (0.80–3.38)	
E	0.89 (0.38–2.10)		1.44 (0.60–3.44)	
Vitreous seeds at first visit		0.031		0.269
No	1		1	
Yes	2.06 (1.07–3.97)		0.75 (0.45–1.25)	
Subretinal seeds at first visit		0.492		0.598
No	1		1	
Yes	1.40 (0.53–3.69)		1.23 (0.57–2.64)	
Germline mutation		0.834		0.891
Negative	1		1	
Positive	1.08 (0.54–2.13)		1.04 (0.58–1.87)	
Treatment naive		0.91		0.673
No	1		1	
Yes	1.03 (0.58–1.84)		1.12 (0.66–1.88)	
Intraoperative pressure hypertensive		0.058		0.281
No	1		1	
Yes	2.17 (0.97–4.85)		1.33 (0.79–2.23)	
Maximum melphalan dose	1.14 (0.97–1.34)	0.115	0.99 (0.94–1.05)	0.783
Maximum topotecan dose	1.61 (0.96–2.69)	0.07	0.99 (0.93–1.05)	0.687
Maximum carboplatin dose	1.01 (1.00–1.02)	0.07	0.99 (0.98–1.01)	0.325
Cumulative melphalan dose	1.05 (1.01–1.08)	0.006	0.99 (0.95–1.03)	0.615
Cumulative topotecan dose	1.16 (0.99–1.37)	0.073	1.00 (0.95–1.05)	0.917
Cumulative carboplatin dose	1.00 (1.00–1.01)	0.102	1.00 (0.99–1.00)	0.442
OAC^e^ interval (weeks)	1.03 (1.00–1.05)	0.025	1.03 (1.01–1.05)	0.01
OAC^e^ interval categories		0.001		0.096
>37 weeks	1		1	
>24 to < = 37 weeks	0.32 (0.16–0.64)		0.51 (0.28–0.94)	
< = 24 weeks	0.18 (0.05–0.65)		0.60 (0.14–2.65)	
Greater than 3 infusions		0.311		0.895
No	1		1	
Yes	1.51 (0.68–3.34)		0.96 (0.50–1.82)	
Greater 50% OA^e^		< .001		< .001
No	1		1	
Yes	0.33 (0.18–0.59)		0.37 (0.22–0.60)	
First treatment OA^e^		0.057		< .001
No	1		1	
Yes	0.44 (0.19–1.03)		0.34 (0.20–0.56)	
Number of drugs		0.027		0.305
1	1		1	
2	2.38 (1.10–5.13)		4.92 (0.65–37.50)	
3	2.60 (1.27–5.29)		4.32 (0.59–31.53)	
Bridge		0.014		0.337
No	1		1	
Yes	2.50 (1.21–5.18)		1.42 (0.69–2.91)	
International Bridge		NA		0.981
No			1	
Yes	NA		1.01 (0.56–1.80)	

^a^HR = hazard ratio

^b^CI = confidence interval

^c^RE = Reese-Ellsworth

^d^ICRB = International Classification of Retinoblastoma

^e^OAC = ophthalmic artery chemosurgery/ OA = ophthalmic artery

**Fig 4:**
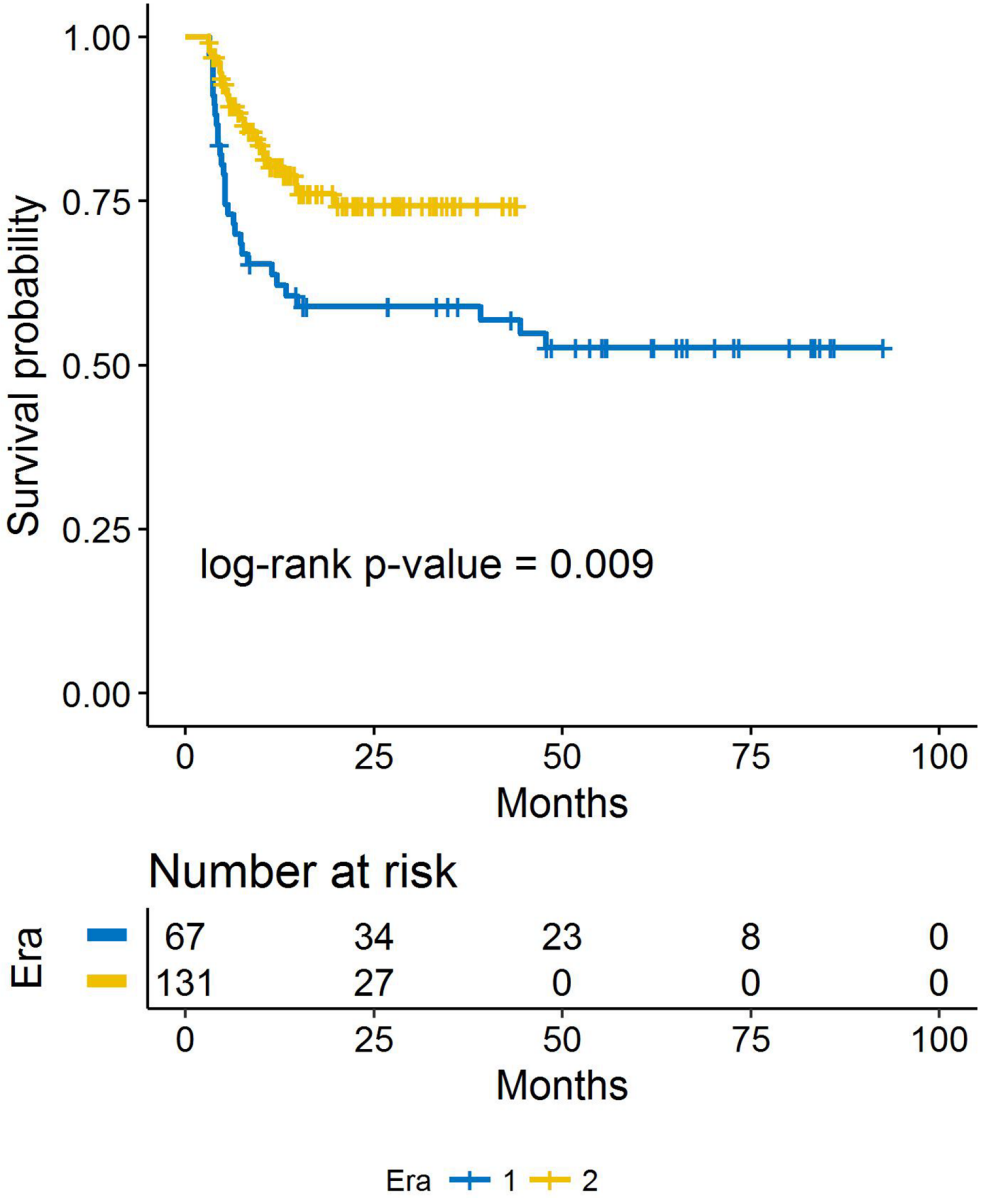
Kaplan-Meier estimates of recurrence-free survival by era, among eyes that had vitreous seeds.

Among patients with vitreous seeds, 1-year RFS increased from 63.8% (95% CI 53.2–76.5%) in Era 1 to 80.2% (95% CI 73.1–88.1%; p-value 0.009) in Era 2. (Fig 4)

### Conditional recurrence-free survival

If a patient was recurrence-free at the end of one year (post-OAC treatment course), then the patient’s estimated RFS was 92% (95% CI 88–96%) at 2 years and 87% (95% CI 82–93%) at 5 years. (Table 5)

**Table 5:** Conditional probability of being recurrence free a certain number of years from the end of treatment.

	Probability of surviving to certain number of years from end of treatment (95% CI)				
Number of years from end of treatment	1	2	3	4	5
0	0.76 (0.72–1.00)	0.70 (0.65–1.00)	0.70 (0.65–1.00)	0.67 (0.61–1.00)	0.67 (0.61–1.00)
1	--	0.92 (0.88–0.96)	0.92 (0.88–0.96)	0.87 (0.82–0.93)	0.87 (0.82–0.93)
2	--	-	1.00 (1.00–1.00)	0.95 (0.91–1.00)	0.95 (0.91–1.00)
3	--	--	--	0.95 (0.91–1.00)	0.95 (0.91–1.00)
4	--	--	--	--	1.00 (1.00–1.00)

The row shaded represents traditional Kaplan-Meier survival estimates at baseline

### Treatment-free survival

There were 436 eyes eligible for analyses of treatment-free survival (TFS). Median follow-up time among those who did not receive treatment for recurrent or persistent disease was 23.6 months (min = 3.2, max = 119.7). During that time, 140 eyes received treatment for recurrent or persistent disease. Estimates of 1-year TFS were 68% (95% CI 61–76%) in Era 1 and 73% (95% CI 68–79%) in Era 2.

### Secondary outcomes

**Fig 5:**
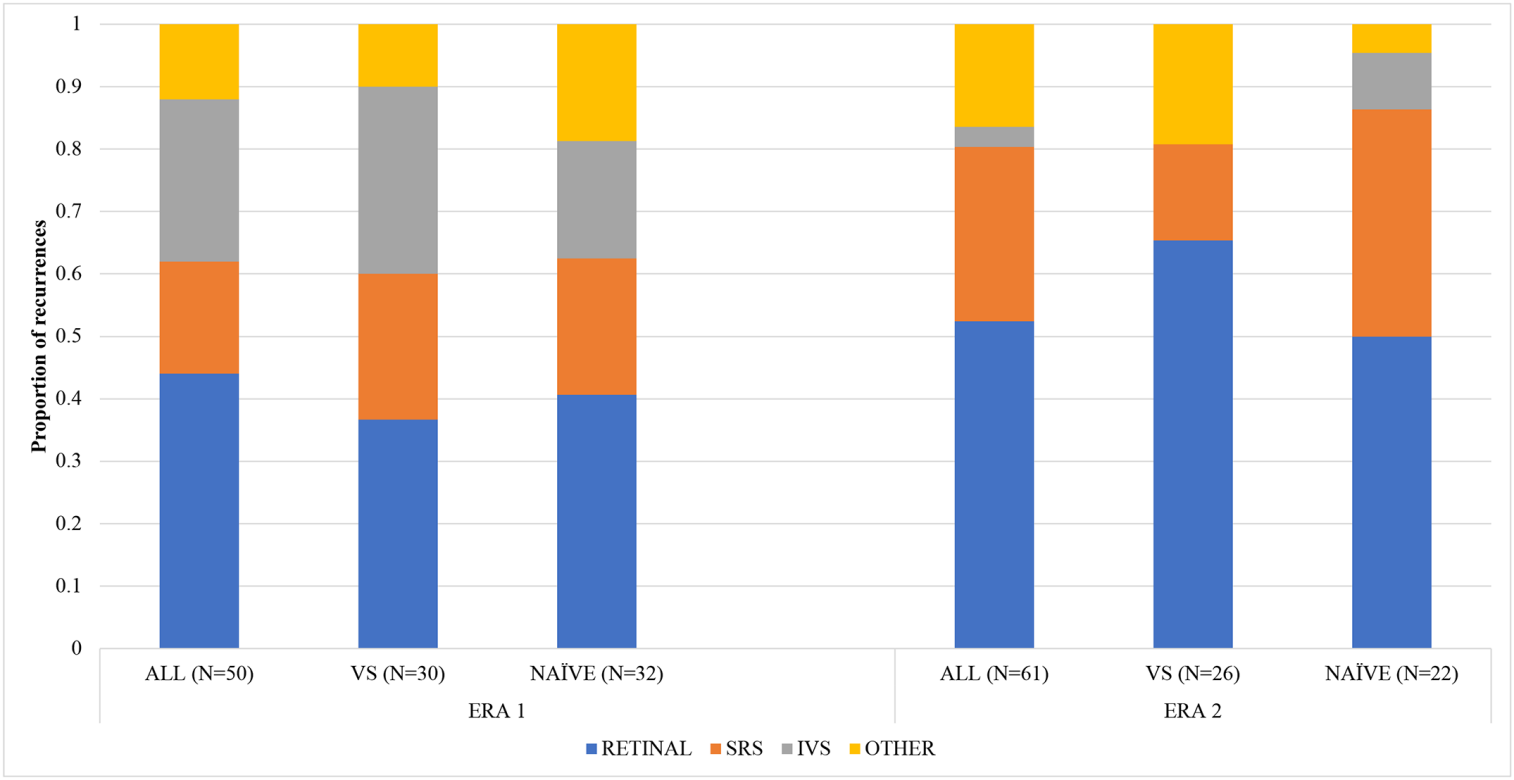
Recurrences by type.

There were 50 recurrences in Era 1 (Fig 5): 22 due to retinal tumors, 13 due to vitreous seeding, 9 due to subretinal seeding, and 6 other (e.g. identified on ultrasound or enucleation pathology). In Era 1, there were two deaths (both due to trilateral disease), two cases of metastasis, and three secondary cancers (one orbital osteosarcoma; two pineoblastomas).

There were 61 recurrences in Era 2: 32 due to retinal tumors, 2 due to vitreous seeding, and 17 due to subretinal seeding. In Era 2, there were four deaths (two due to trilateral disease, one due to metastatic retinoblastoma, and one nonaccidental trauma), three cases of metastasis, and four secondary cancers (one neuroblastoma; three pineoblastomas).

## Discussion

This present study provides two important insights regarding retinoblastoma eyes treated with ophthalmic artery chemosurgery: 1) confirms the high ocular salvage rate and 2) introduces a proposed definition and results regarding recurrent disease and contributing factors.

In this cohort of eyes, the majority of which had advanced disease (64% RE V and 67.2% ICRB D or E), the 1-year Kaplan-Meier estimate for ocular survival was 96%. With the inclusion of enucleated eyes that failed *initial* OAC, the ocular survival was 92%. Finally, using PFS as a measure (following the published standard of including initial failures and events of subsequent enucleation, EBR and OAC from initial visit), the event-free survival was 91%. These are staggering numbers compared to historical rates for eyes treated with other modalities. [11] With the adoption of OAC as first-line treatment for retinoblastoma, eyes that were once destined for removal are being saved, and enucleation rates at our institution are presently at an all-time historical low. [11]

Given the potential of intravitreous chemotherapy to influence outcome, the analysis was divided into era 1 and era 2, with the latter eyes receiving intravitreous chemotherapy if indicated. Both groups had similar classifications and vitreous seed status at presentation. However, a higher percentage of eyes in era 2 were hypertensive, possibly reflecting more advanced disease. Era 1 eyes received more adjunctive cryotherapy and plaque brachytherapy, and era 2 eyes received higher doses of cumulative topotecan and carboplatin during their initial OAC. Fewer era 2 eyes failed initial treatment (7.3% era 1 versus 1.1% era 2), suggesting the combination of more intensive OAC with concomitant intravitreous chemotherapy might result in greater initial success compared with OAC with cryotherapy and brachytherapy.

The present study analyzed recurrence-free survival, made possible by high ocular survival rates. Recurrence-free survival is not a frequently reported outcome in retinoblastoma studies, which made this a pioneering goal in several respects. First, the dearth of reported recurrence rates limits comparisons with historical treatments. In the few studies with data, the rate of recurrence is generally at least 40%, although restriction to one group of eyes or relatively outdated management makes it difficult to draw meaningful comparisons. [12, 13] Secondly, there is no standard by which recurrence is defined in retinoblastoma. Even in the present literature, if recurrent disease is the main topic of the study, it is rarely defined. [12–14] The definition presented in this paper may serve as a standard for future studies.

Overall, approximately a quarter of the eyes developed recurrent disease, the majority within the first year. Of eyes that recurred, 54% were treated for recurrence with focal methods alone (laser, cryotherapy, brachytherapy, intravitreous chemotherapy), 29% required only laser or cryotherapy, and 30% of eyes received additional OAC. Eyes that were free of recurrence after the first year had only an 8% risk of developing recurrent disease by two years. Similarly, one previous study reported all recurrences occurred within the first year following OAC, suggesting that careful surveillance of eyes is particularly important in this time frame. [15]

Recurrence estimates at 1 year were not statistically different between era 1 and era 2. However, even though vitreous seeds at presentation were similar between era 1 and era 2, era 1 eyes recurred more: the 1-year RFS increased from 63.8% in era 1 to 80.2% in era 2. Finally, more eyes in era 1 had recurrent *vitreous* disease compared to era 2 (13 eyes versus 2 eyes), suggesting the initial intravitreous chemotherapy delivered in era 2 was instrumental in sterilizing the vitreous cavity. This is consistent with prior findings. [14]

Two striking factors were associated with better RFS. First, in both eras, eyes recurred significantly less if greater than 50% of the OAC infusions were delivered via the ophthalmic artery as opposed to alternate routes. It is presumed that the more direct delivery allows for a higher concentration of drug to the eye. Second, for both eras, eyes receiving OAC infusions greater than 4 weeks apart (defined as >37 days compared to less than or equal to 37 days) were significantly more likely to recur. Longer intervals between treatments might indicate a general level of non-compliance to treatment, which might confound these results. However, it suggests that maintaining the intended target of delivering OAC infusions 3–4 weeks apart is important for minimizing the potential for recurrent disease.

Many factors were not associated with worse recurrence, including the presence of subretinal seeds at presentation. This is in contrast to eyes treated with systemic chemotherapy, in which subretinal seeds are the most important factor in predicting recurrent disease. [13] Surprisingly, factors predictive of disease advancement (classification, receiving greater than three OAC infusions at initial treatment, ocular hypertension) were not associated with RFS. One would expect prior treated eyes to potentially be resistant to subsequent treatments and at risk for recurrent disease. Finally, patients with bilateral disease receiving tandem OAC infusions (bilateral infusions during the same session) often exceed the maximum systemic melphalan dose (.4mg/Kg) in one eye, thereby limiting the allotted melphalan that can be delivered to the fellow eye. [16] Despite this relative limitation, bilateral disease was not associated with worse RFS.

In summary, the important findings for practicing clinicians are as follows: 1) Approximately a quarter of eyes initially treated with ophthalmic artery chemosurgery might develop recurrent disease, and despite this, have a very good chance of being saved. 2) The majority of recurrences occur in the first year following initial completion of OAC, and therefore careful surveillance in the first year is encouraged. 3) Eyes that receive the majority of its drug infusions via non-ophthalmic artery routes appear to be at higher risk for recurrence and might warrant close monitoring. 4) Initial OAC appears to be associated with more recurrence if delivered greater than 4 weeks apart, and therefore an interval of less than 4 weeks apart is preferred. 5) In eyes with vitreous seeds at presentation, intravitreal injections are useful in minimizing future vitreous recurrence.

Measurability of treatment success is important in future treatment advancements and consideration of recurrence-free survival appears to be useful. With patient survival over 99% and ocular survival surpassing 90%, the next advancement in the management of retinoblastoma entails reducing instances of recurrent disease and the time, money, and potential toxicity they afford.
